# Influence of the Silica Specific Surface Area and Ionic Liquids on the Curing Characteristics and Performance of Styrene–Butadiene Rubber Composites

**DOI:** 10.3390/ma14185302

**Published:** 2021-09-14

**Authors:** Anna Sowińska-Baranowska, Magdalena Maciejewska

**Affiliations:** Department of Chemistry, Institute of Polymer and Dye Technology, Lodz University of Technology, Stefanowskiego Street 16, 90-537 Lodz, Poland

**Keywords:** vulcanization, rubber, silica, ionic liquids, cure characteristics, thermal behavior, mechanical properties

## Abstract

In this work, we present the effect of silica’s specific surface area (180 m^2^·g^−1^ and 380 m^2^·g^−1^, respectively) on the crosslinking of styrene–butadiene rubber (SBR) composites, as well as their crosslink density and functional properties, such as thermal stability, damping behavior, resistance to thermo-oxidative aging, and tensile properties. Ionic liquids (ILs) with a bromide anion and different cations, i.e., 1-butyl-3-methylimidazolium (Bmi), 1-butyl-3-methylpyrrolidinium (Bmpyr), and 1-butyl-3-methylpiperidinium (Bmpip), were used to enhance the cure characteristics of SBR compounds and the functional properties of SBR vulcanizates. It was proven that apart from the silica’s specific surface area, the filler–polymer and filler–filler physical interactions have a significant impact on the vulcanization kinetics of silica-filled SBR composites. Additionally, the performed studies have shown that ILs positively affected the dispersion of silica’s particles and reduced their ability to form agglomerates in the elastomer matrix, which enhanced the functional properties of the SBR vulcanizates.

## 1. Introduction

Most elastomers are unviable for industrial applications without any reinforcing filler, since they do not meet the technological requirements for, e.g., mechanical strength, abrasion resistance, damping performance, or hardness. Therefore, in the rubber industry, many types of fillers with different characteristics and activities are used [1,2,3,4,5]. The reinforcing effect of the filler mainly depends on the size of the particles and their distribution in the elastomer matrix, as well as on the specific surface area and chemical and physical nature of the filler [6]. As far as elastomer composites are concerned, it should be noted that fillers can have a significant impact on the course and efficiency of vulcanization, which is one of the most important processes in elastomer technology. The surface of fillers, particularly silica, can adsorb accelerators, which deteriorates their effectiveness and consequently results in the extension of the vulcanization time of rubber compounds and a reduction in the crosslink density of the vulcanizates [7,8,9,10]. Furthermore, the adsorption of the crosslinking system on the filler’s surface results in a heterogeneous distribution of crosslinks in the elastomer network, since the area directly around the filler particles is crosslinked in a higher degree compared to the regions of the elastomeric network, which are not located in the immediate vicinity of the filler particles. The heterogeneity of elastomer networks strongly affects the mechanical properties of the vulcanizates [6]. Therefore, to ensure efficient vulcanization and satisfactory vulcanizate performance, an attempt must be made to reduce the susceptibility of the filler surface to the adsorption of the curatives.

Rubber compounds generally contain about 30% by weight of active fillers such as silica and carbon black [11,12]. Silica is a non-black reinforcing filler [13]. Its surface consists of a large amount of siloxane and polar silanol functional groups, and the silanol or hydroxyl groups are acidic [14]. The application of silica in rubber compounds has some difficulties, i.e., incompatibility with nonpolar rubbers due to the highly polar nature of silica, which results in non-homogenous dispersion and distribution of the silica particles in the elastomer matrix, and therefore poor filler–rubber interactions [15]. The large amount of polar silanol groups (Si-OH) on the silica’s surface results in strong particle–particle interactions via hydrogen bonds, which causes a high ability of this filler to agglomerate in the rubber matrix. Furthermore, due to its surface polarity and hydrophilicity, silica has a strong ability to adsorb moisture. The amount of water adsorbed on the surface of silica controls the ionization of the hydroxyl groups [16], which has an unfavorable influence on the cure characteristics of rubber compounds [14] and silica’s interactions with the elastomer matrix. In addition, the acidic character of silica induces some interactions with basic vulcanization accelerators, deteriorating the curing characteristics due to the unacceptably long cure times and slow cure rates, and consequently decreasing the crosslinking efficiency of the sulfur curing systems. These properties lead to numerous difficulties when silica is used in rubber products. In order to overcome these inconveniences by reducing the ability of silica particles to agglomerate and their tendency to adsorb curatives, a wide range of additives has been applied, e.g., silanes, ionic liquids (ILs), and surfactants [13,17,18,19,20,21,22]. 

A common approach to improve the dispersion of silica in the elastomer matrix concerns the application of silanes. Ansarifar et al. [14] examined the effect of the increased level of bifunctional organosilane, i.e., bis[3-(triethoxysilyl)propyl] tetrasulfide (TESPT) used with precipitated silica, on the Mooney viscosity and curing behavior of NR composites. The addition of TESPT significantly reduced both the Mooney viscosity and the optimal vulcanization time of the rubber compounds, and so it beneficially affected the cure characteristics and processability of silica-filled NR compounds. The same organosilane was used by Castellano et al. [20] to modify the surface of amorphous precipitated silica in chloromethane solution. Owing to the silanization, the silica particles exhibited a lower tendency to interact with each other, and consequently a higher dispersibility of the filler’s particles in the elastomer matrix was achieved. This resulted in the enhanced mechanical properties of the vulcanizates. Mora-Barrantes et al. [23] performed the silanization of fumed silica using different bifunctional organosilanes as coupling agents, i.e., 3-octanoylthio-1-propyltriethoxy silane, mercaptopropyl trimethoxy silane, and TESPT. The applied silanes bore sulfur-based reactive groups, which were able to interact with rubber macromolecules during vulcanization. The application of the silanized fumed silica in the styrene–butadiene rubber composites significantly improved their mechanical properties due to the better miscibility of the modified silica nanoparticles with the elastomer and consequently homogeneous dispersion of the filler in the elastomer matrix.

To reduce the ability of silica particles to agglomerate in the elastomer matrix and consequently to enhance the performance of final rubber products, various modifications of the silica surface have been performed, e.g., IL immobilization and plasma surface modification [19,20,21]. Furthermore, Guo et al. [22] adopted sorbic acid (SA) as a novel modifier to facilitate the dispersion of silica in the SBR matrix and to enhance the interfacial filler–elastomer matrix interactions. Strong interfacial bonding between the silica and the rubber matrix occurred, which was proven to result from the sorbic acid intermediated linkages. Consequently, the dispersion of the silica in the elastomer matrix and further the mechanical performance of SBR/silica compounds were remarkably improved.

Currently, ILs are becoming more and more popular as additives improving the dispersion of fillers, including silica, in the elastomer matrix. In our previous work [17], the influence of silica and ILs on the crosslinking and performance of natural rubber (NR) bio-composites was established. Silica strongly affected the cure characteristics of NR composites by increasing the viscosity of the uncured rubber compounds, enhancing the torque increment during rheometric measurements and significantly extending the optimal vulcanization time as compared to the unfilled NR. ILs improved the cure characteristics of NR compounds as well as the crosslink density and mechanical performance of the vulcanizates. A beneficial influence of ILs on the curing characteristics and performance of elastomers was confirmed by Hussain et al. for silica-filled butadiene rubber [24] and by Zhang et al. for silica-filled NR nanocomposites [25]. Lei et al. [26] developed a novel functional IL, i.e., 1-methylimidazolium methacrylate (MimMa), which was applied for modifying styrene–butadiene rubber (SBR)/silica composites. MimMa significantly enhanced the interfacial filler–elastomer interactions and consequently the mechanical properties of SBR vulcanizates due to the improved silica dispersion induced by MimMa. A hydrogen bonding between the silica and the ions of MimMa was proven.

Thus, taking into account that ILs facilitate the filler’s dispersion [27,28], in this work we applied ILs with different cations and a bromide anion to decrease the ability of the silica to agglomerate in the elastomer matrix and to adsorb the curing system. In this way, we wanted to establish whether ILs may be an alternative to the commonly used silanes. Having known that the reinforcement effect of fillers and their ability to agglomerate in the elastomer matrix strongly depend on the filler’s specific surface area, we studied the influence of this property on the activity of silica in the SBR composites. Two types of silica significantly differing in the specific surface area (180 m^2^·g^−1^ and 380 m^2^·g^−1^, respectively) were applied as fillers and their impact on the cure characteristics and the performance of SBR composites was explored. The aim of this study was also to explore the influence of the ILs’ structure, i.e., the type of cation, on the vulcanization and performance of the silica-filled SBR composites.

## 2. Materials and Methods

### 2.1. Materials

**Figure 1:**
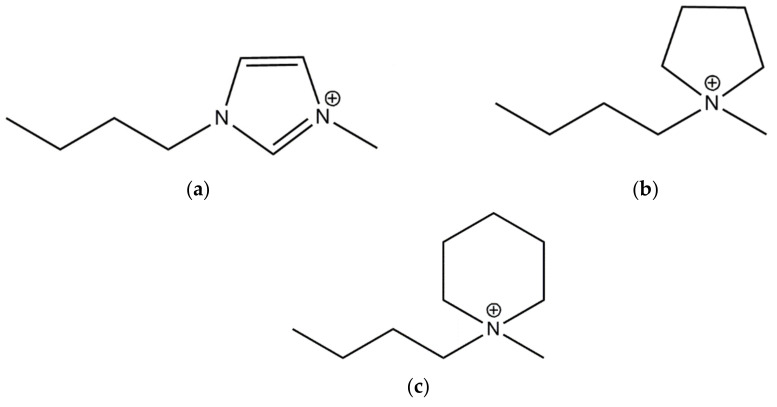
Structure of IL cations: (**a**) 1-butyl-3-methylimidazolium; (**b**) 1-butyl-1-methylpyrrolidinium; (**c**) 1-butyl-1-methylpiperidinium.

A standard grade styrene–butadiene rubber (SBR, KER 1500 type), with approximately 23.5 wt.% bonded styrene, was purchased from Synthos SA (Oswiecim, Poland). It was characterized by a Mooney viscosity of ML1+4 (100 °C): 50 MU. Two types of silica with different specific surface areas, i.e., AEROSIL^®^ 380 (A380, specific surface area of 380 m^2^·g^−1^, pH 3.7–4.5, purity ≥99.8%) and ULTRASIL VN3 (VN3, specific surface area of 180 m^2^·g^−1^, pH 6.5, purity ≥97.0%), were provided by Evonik Industries (Essen, Germany) and applied as fillers. SBR compounds were cured using conventional curing system containing sulfur having a purity of 99.9% (Siarkopol, Tarnobrzeg, Poland), which was employed as the curing agent, 2-mercaptobenzothiazole (MBT, purity 97.0%) and N-cyclo-hexyl-2-benzothiazolesulfenamide (CBS, purity 98.0%), which were applied as vulcanization accelerators (Sigma-Aldrich, Schelldorf, Germany), and microsized zinc oxide (ZnO) with a specific surface area of 10 m^2^·g^−1^ and purity 99.0% (Huta Bedzin, Bedzin, Poland), which was used to activate the vulcanization. Additionally, three ionic liquids (ILs) with characteristics presented in Table 1 were employed (IoLiTec Ionic Liquids Technologies GmbH, Heilbronn, Germany) to enhance the dispersibility of the silica in the elastomer matrix and for the adsorption of curatives. These ILs consisted of bromide anion and different cations with butyl substituents. The structures of IL cations are presented in Figure 1.

### 2.2. Preparation and Characterization of SBR Compounds Filled with Silica

SBR compounds were prepared in a two-step procedure, exploiting a laboratory two-roll mill (David Bridge & Co., Rochdale, UK, Country) with the following roll dimensions: D = 200 mm, L = 450 mm. The friction and the width of the gap between rollers were 1–1.2 mm and 1.5–3 mm, respectively, whereas the rotational speed of the front roll was 16 min^−1^. The average temperature of the rolls during preparation of rubber compounds was approximately 30 °C. First, the reference unfilled rubber compound was prepared (R0), and then two masterbatches filled with an appropriate silica and containing sulfur and vulcanization accelerators were manufactured. Next, each masterbatch was divided into four equal pieces and the vulcanization activator (ZnO) without IL was added to one of these pieces (R1–R2). The general recipes of the reference SBR compounds are given in Table 2. Then, ZnO previously mixed with a proper IL was added to the rest of the pieces (the rubber compounds were marked as IL1–IL6). The general formulations of SBR compounds containing ILs are presented in Table 3 as parts per hundred of rubber (phr).

SBR compounds were cured at 160 °C, at 15 MPa pressure using the optimal vulcanization times determined during rheometric tests. The cure characteristics of SBR compounds were explored at 160 °C according to the ISO 6502 [29] standard procedures using the rotorless D-RPA 3000 rheometer (MonTech, Buchen, Germany). Rheometric tests were performed for three pieces of each rubber compound with a mass of approximately 5 g. The optimal vulcanization time (t_95_) corresponds to the end of the curing stage and relates to the time needed for the cured rubber to achieve optimal properties. Moreover, the optimal vulcanization time is recognized as the time necessary to achieve the optimum network density of the vulcanizates [30]. It was determined as the time for rheometric torque to reach 95% of the maximum achievable torque value (*S*_95_), which in turn was given by Equation (1) [31], where ∆*S* is the torque increase during rheometric test, calculated as the difference between the maximum (*S_max_*) and minimum (*S_min_*) torque. Adapting the similar equation, *S*_05_ torque was calculated, which was used to determine the scorch time (t_05_).

(1) $$S_{95}=0.95∆S+S_{min},$$

For examining the effect of the silica’s specific surface area and ILs on the temperatures and the enthalpy of SBR curing reactions, a differential scanning calorimeter DSC1 (Mettler Toledo, Greifensee, Switzerland) equipped with STARe software (Version 10, 2010, Greifensee, Switzerland) was employed. The onset temperature of the peak referring to the curing reactions was established according to the procedure given in the ISO 11357-1 [32] standard. Nitrogen (flow rate 80 mL/min) was used as the protective gas, whereas liquid nitrogen was applied to cool the sample before the measurement. The analysis was carried out for a small pieces of rubber compounds with a mass of approximately 10 mg, which were placed in a hermetically sealed aluminum crucible with a capacity of 40 µL and heated from −100 °C to 250 °C, with a constant heating rate of 10 K/min. 

According to the standard ISO 1817 [33], the crosslink density of SBR vulcanizates filled with silica was established by their equilibrium swelling in toluene and calculated using the Flory–Rehner equation [34]. Small pieces of the vulcanizates with masses in the range of 20–30 mg were swollen in toluene for 48 h at room temperature. Then, after removing the solvent and weighing the samples, they were dried at 50 °C for another 48 h. Finally, the dried samples were reweighed. Four pieces differing in shape were used for the swelling tests for each vulcanizate. First, the equilibrium swelling *Q_w_* of the vulcanizates was calculated using Equation (2):

(2) $$Q_{w}=\frac{m_{sw}-m_{d}}{m_{d}},$$

where *m_sw_* is the mass of the swollen sample, and *m_d_* is the mass of the swollen sample after drying. Then, the reduced equilibrium swelling *Q_r_* for the vulcanizates containing filler was calculated with Equation (3):

(3) $$Q_{r}=Q_{w}\sum \frac{m_{i}}{m_{f}},$$

where *m_i_* is the total mass of the rubber compound, and *m_f_* is the total mass of mineral substances in the rubber compound including filler. Next, the volume fraction of rubber in the swollen gel *V_r_* was calculated with Equation (4):

(4) $$V_{r}=\frac{1}{1+Q_{r}\frac{\rho_{r}}{\rho_{s}}},$$

where *V_r_* is the volume of the elastomer fraction in swollen gel, *ρ_r_* is the density of the rubber, and *ρ_s_* is the density of the solvent. 

Finally, based on the Flory–Rehner equation [34] (Equation (5)), the crosslink density *ν_t_* was calculated as follows:

(5) $$\nu_{t}=\frac{ln\left(1-V_{r}\right)+V_{r}+\mu V_{r}^{2}}{V_{0}\left(V_{r}^{\frac{1}{3}}-\frac{V_{r}}{2}\right)},$$

The Huggins parameter of elastomer–solvent (SBR–toluene) interaction (*χ*) given by Equation (6) [35] was applied for calculations:

(6) $$\chi =0.37+0.56V_{r},$$

Thermogravimetry (TG) was employed to study the thermal stability of silica, ILs, and finally SBR vulcanizates. Thermogravimetry/differential scanning calorimetry TGA/DSC1 analyzer (Mettler Toledo, Greifensee, Switzerland) was employed to conduct measurements for the samples with a mass of approximately 10 mg placed in the open alumina crucibles with a capacity of 70 µL. TG analysis of pure fillers, i.e., silica Ultrasil VN3 and Aerosil 380, and ILs was carried out in the temperature range of 25–600 °C in argon atmosphere (50 mL/min) with a heating rate of 10 K/min. On the other hand, TG measurements for SBR vulcanizates were performed using a two-step procedure. First, small pieces of the vulcanizates were heated in the temperature range of 25–600 °C, with a heating rate of 20 K/min in an argon atmosphere (50 mL/min). Then, the gas was changed into air (50 mL/min), and heating was continued up to 800 °C with the same heating rate. 

The mechanical properties of SBR vulcanizates were investigated according to the ISO-37 [36] standard procedures using a Zwick Roell 1435 (Ulm, Germany) universal testing machine. Tensile tests were carried out in static conditions for six dumb-bell shaped samples with a thickness of approximately 1 mm and the width of the measuring section of 4 mm. The crosshead speed during tensile tests was 500 mm/min. 

The hardness of three disc-shaped SBR vulcanizates was examined using Shore’s method following the standard ISO 868 [37] by Zwick Roell 3105 (Ulm, Germany) hardness tester. The hardness was measured at five places on each of the discs distant by at least 6 mm apart.

The thermo-oxidative aging of SBR vulcanizates was performed according to the ISO 188 standard [38]. Plates of the vulcanizates with a thickness of approximately 1 mm were stored in a drying chamber (Binder, Tuttlingen, Germany) at 100 °C for seven days. To determine the resistance of the elastomers to aging, their mechanical properties, hardness, and crosslink densities were explored and compared with the values obtained for non-aged vulcanizates. To quantify the resistance of elastomers to prolonged thermo-oxidation, the aging coefficient (*AF*) was calculated according to Equation (7) [39,40], where *TS* is the tensile strength of vulcanizates and *E_b_* is the elongation at break.

(7) $$AF=\frac{{\left(E_{b}\times TS\right)}_{after\;aging}}{{\left(E_{b}\times TS\right)}_{before\;aging}},$$

Dynamic mechanical analysis (DMA) was carried out in tension mode employing a DMA/SDTA861e analyzer (Mettler Toledo, Greifensee, Switzerland). Analysis of the vulcanizates was conducted in the temperature range of −100 to 70 °C with a heating rate of 3 K/min, a frequency of 1 Hz, and a strain amplitude of 4 µm. The temperature of the elastomer glass transition (T_g_) was indicated from the maximum of the tan δ = f(T) curve, where tan δ is the mechanical loss factor, and T is the measurement temperature.

Scanning electron microscopy (SEM) images of SBR vulcanizates were taken using an LEO1450 scanning electron microscope (Carl Zeiss AG, Oberkochen, Germany). Prior to the analysis, samples of the vulcanizates were broken down using liquid nitrogen. Next, their fractures were coated with a carbon layer and explored. SEM images were taken to estimate the dispersion of the silica and curing system particles in the elastomer matrix.

## 3. Results and Discussion

### 3.1. Thermal Stability of Silica and Ionic Liquids

**Figure 2:**
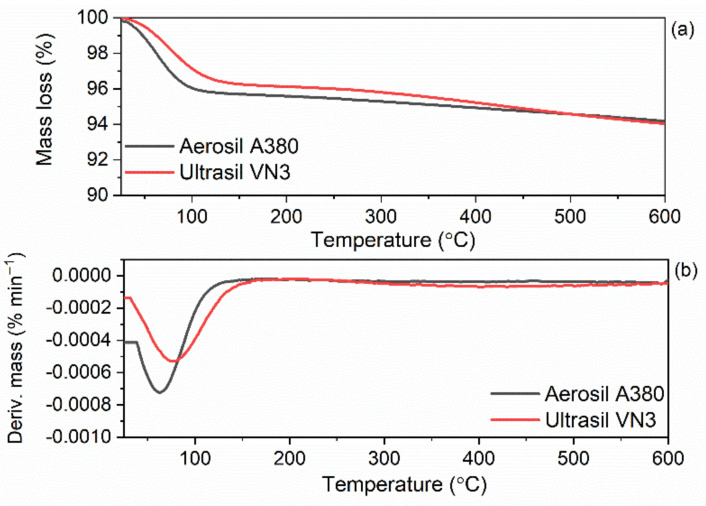
Thermogravimetric (TG) and derivative thermogravimetric (DTG) curves of silica A380 and VN3: (**a**) TG curves; (**b**) DTG curves.

In the first step of the studies, thermogravimetric analysis (TGA) was used to examine the thermal stability of the silica Aerosil 380 and Ultrasil VN3. Additionally, the derivative thermogravimetric (DTG) peak temperature (T_DTG_) was established as the temperature at which the rate of the sample decomposition was the highest. TG and DTG curves for A380 and VN3 silica are presented in Figure 2.

Silica A380 and VN3 demonstrated a similar thermal behavior. On the TG curve corresponding to the thermal decomposition of silica A380, a mass loss of approximately 6.4% occurred at a temperature below 150 °C, while the DTG peak temperature related to this process was at 65 °C. This was due to the desorption of moisture, as silica is a highly hygroscopic powder [41]. Similar results were obtained in our previous work [24]. Regarding silica VN3, the mass loss corresponding to the moisture desorption was approximately 3.7% and the temperature of the DTG peak was 73 °C. The next mass loss obtained for both fillers was approximately 2–3% in the temperature range of 350–600 °C and resulted from dehydroxylation of the silica surface [42]. As expected, silica A380 with a significantly higher specific surface area exhibited remarkably higher ability to adsorb moisture, as was confirmed by a significantly higher mass loss at a temperature below 150 °C determined for A380.

**Figure 3:**
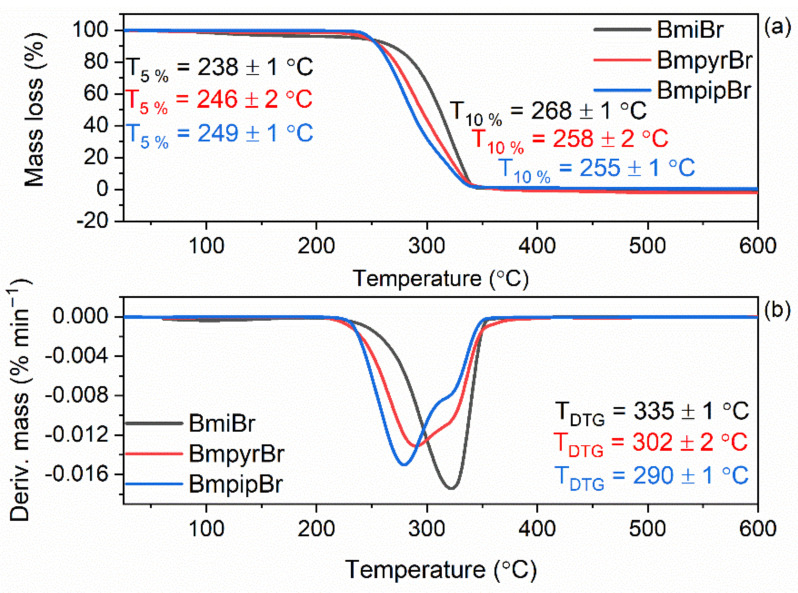
Thermal stability of BmiBr, BmpyrBr, and BmpipBr: (**a**) TG curves; (**b**) DTG curves.

The thermal stability of ionic liquids (ILs) is well-known to depend on the type of cation and anion. The examined ILs consist of the same bromide (Br) anion but bear cations with different heterocyclic rings with a quaternary nitrogen atom, i.e., alkylimidazolium, alkylpyrrolidinium, and alkylpiperidinium with the same substituent in the form of a butyl chain. Therefore, we intended to explore the influence on the heterocyclic ring in the structure of ILs on their thermal stability. Thermogravimetry (TG) was applied to establish the temperature at 5% mass loss in relation to the initial mass of the IL sample, which corresponds to the onset decomposition temperature (T_5%_). TG and DTG curves for studied ILs are shown in Figure 3.

Investigated ILs completely decomposed in the temperature range of 25–400 °C. Considering the T_5%_, i.e., the onset decomposition temperature, the imidazolium IL (BmiBr) showed the lowest degradation temperature (T_5%_ of approximately 238 °C) as compared to the pyrrolidinium IL, i.e., BmpyrBr (T_5%_ of approximately 246 °C) and the piperidinium one (BmpipBr), which exhibited the highest T_5%_ of approximately 249 °C. On the other hand, the temperature at 10% mass loss (T_10%_) for BmpipBr was approximately 3 °C and 10 °C lower than for BmpyrBr and BmiBr, respectively. Moreover, analyzing the TG and DTG curves, it was observed that the main stage of BmpipBr thermal decomposition also started at a lower temperature compared to other ILs, especially BmiBr. Therefore, piperidinium IL showed the lowest temperature of the maximum mass loss rate, which proved that BmpipBr had the lowest thermal stability among the tested ILs. Referring to T_10%_ and T_DTG_ temperatures, the most thermally stable seemed to be the imidazolium IL, i.e., BmiBr. The thermal decomposition of the imidazolium and pyrrolidinium ILs was thoroughly described in our previous work [17]. In this work, we confirmed that the thermal stability of ILs having the same anion depends on the cation structure, and more precisely on the type of ring present in the IL. The presence of the unsaturated heterocyclic imidazolium ring in the cation facilitates the thermal stability of ILs compared to those having saturated heterocyclic rings, e.g., pyrrolidinium or piperidinium. The high thermal stability of imidazolium salts compared to ILs with other cations has also been confirmed by Xue et al. [43]. Furthermore, Belhocine et al. [44] have reported that piperidinium and pyrrolidinium ILs with various anions were thermally stable up to 330 °C, which correlates well with our results. Most importantly, the examined ILs were thermally stable at vulcanization temperature, i.e., 160 °C, which is crucial for their applications in rubber technology.

### 3.2. Dispersion of Curatives and Silica in SBR Matrix

**Figure 4:**
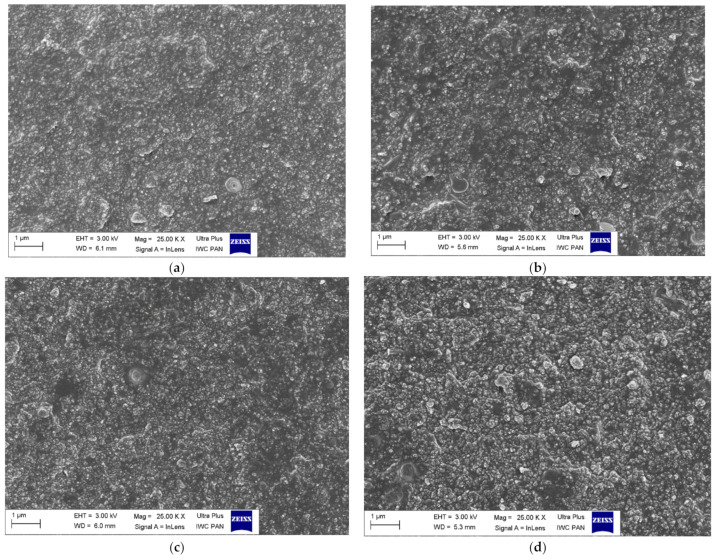
Scanning electron microscopy (SEM) images of SBR vulcanizates: (**a**) A380; (**b**) VN3; (**c**) A380/BmiBr; (**d**) VN3/BmiBr.

The main goal of elastomer technology is to produce a rubber product with the appropriate mechanical performance and hardness. A crucial aspect of the reinforcement effect is producing a homogenous dispersion of the filler particles in the elastomeric matrix, which causes a good interphase adhesion. Moreover, the contact between components of the curing system in the elastomer matrix should be maximized to enhance the crosslinking efficiency. Most solid additives exhibit a high ability to agglomerate in the elastomer matrix, and hence achieving uniform distribution of their particles in the elastomer is technologically difficult. Therefore, SEM images were taken to study the dispersion degree of the silica as well as curatives in the crosslinked SBR matrix. The results for A380 and VN3-filled SBR vulcanizates without ILs and containing BmiBr are shown in Figure 4.

Regardless of the specific surface area, the particles of both silicas were homogeneously dispersed in the elastomer matrix (Figure 4a,b). Silica particles were surrounded by an elastomeric film that penetrated between them, so they exhibited good wettability by the elastomer matrix. However, it should be noted that A380 silica with a significantly higher specific surface area was dispersed in SBR as finer particles compared to VN3. The incorporation of ILs did not significantly affect the dispersion of both silicas’ particles in the SBR matrix (Figure 4c,d). No significant differences between the SEM images of the vulcanizates without ILs and containing BmiBr were observed considering the wettability of silica particles by the SBR elastomer.

### 3.3. Effect of Silica and Ionic Liquids on Cure Characteristics and Crosslink Density of SBR Composites

The rheometric properties of SBR compounds were investigated to determine the influence of the silica’s specific surface area and ILs on the curing parameters. The rheometric measurements were conducted at 160 °C and the results are summarized in Table 4.

As mentioned, since the specific surface area of A380 (380 m^2^·g^−1^) is significantly higher than that of VN3 silica (180 m^2^·g^−1^), the amount of the hydroxyl groups on the filler’s surface is also much higher for A380. Thus, the ability of A380 to adsorb the curatives is expected to be higher compared to VN3 and the influence of this filler on the cure characteristics of SBR is presumed to be more meaningful than that of VN3. This assumption was confirmed by the results of the studies presented in Table 4.

The minimum rheometric torque (*S_min_*) values and, consequently, the torque increase (∆*S*) of SBR compounds filled with A380 were significantly higher compared to the unfilled sample and compounds filled with VN3 silica. The *S_min_* is a measure of the viscosity of the uncrosslinked rubber compound. Higher values of *S_min_* were associated with the size of the nanoparticles and the greater specific surface area of A380 silica, which limited the mobility of the elastomer molecular chains to a higher degree compared to VN3, and consequently significantly increased the viscosity of the uncrosslinked rubber compounds. The same effect of silica A380 on the *S_min_* and ∆*S* was observed in our previous work [17] for NR compounds as well as for NBR [45] and SBR [46] compounds. As expected, VN3 silica also increased the *S_min_* compared to unfilled SBR, but half as much as A380. Thus, SBR compounds filled with VN3 should be easier to process compared to those containing A380. Regardless of their structure, ILs did not significantly affect the viscosity of the uncrosslinked SBR compounds. None of the rubber compounds containing ILs showed a higher *S_min_* value than the samples without ILs.

Rubber compounds filled with silica exhibited significantly higher maximum torque (*S_max_*), and consequently Δ*S*, compared to the unfilled benchmark. This was due to the hydrodynamic effect of filler and filler–filler interactions [47]. It should be noticed that SBR filled with silica A380, which showed a significantly higher specific surface area, demonstrated remarkably higher *S_max_* and Δ*S* compared to rubber compounds containing VN3—silica with the lower specific surface area. Thus, it was concluded that A380 was characterized by a stronger reinforcing effect and elastomer–filler interactions compared to VN3. Moreover, the increase in *S_min_*, *S_max_*, and Δ*S*, respectively, resulted from the introduction of the rigid phase of the filler network into the soft elastomer matrix, which significantly reduced the mobility of elastomer chains. The stiffness of SBR filled with A380 was observed to be considerably higher compared to VN3-containing rubber compounds. Taking into account the measurement error, ILs did not meaningfully alter the values of *S_max_* and Δ*S* of SBR compounds.

Regardless of the silica used, SBR compounds exhibited a scorch time (t_05_) twice as long as the unfilled sample, so the addition of silica reduced the risk of scorching at 160 °C. A similar effect should also be expected at the lower temperatures at which SBR compounds are commonly processed. ILs decreased the t_05_ to a value similar to that obtained for the unfilled SBR. Regarding the optimal vulcanization time (t_95_*)*, a strong influence of the silica’s specific surface area on this parameter was indicated. The unfilled SBR compound showed a t_95_ of 3 min. Both silicas extended the t_95_ of SBR compounds: VN3 to 10 min, while A380 up to 42 min, respectively. This was due to the adsorption of curatives onto the silica surface, which is associated with the decreased activity of the crosslinking system [7,8,48]. The greater specific surface area of A380 silica and consequently the higher content of active centers, i.e., silanol groups on its surface, compared to VN3 resulted in the stronger adsorption of the curing system. Furthermore, sulfur vulcanization prefers an alkaline environment to proceed faster [49]. The silicas used are characterized by a different pH. Silica VN3 has a pH of about 6.5, which is almost neutral, while A380 exhibits a pH in the range of 3.7–4.5, which is acidic. Hence, the incorporation of 30 phr of acidic silica increases the acidity of the vulcanization environment, contributing to the reduction in its efficiency. These theses were confirmed by the results of vulcanizates’ crosslink density determined by the equilibrium swelling method. Regarding vulcanizates without ILs, the A380-filled vulcanizate exhibited significantly lower crosslink density compared to the unfilled one and that containing VN3. Similarly, after adding ILs, vulcanizates filled with VN3 showed higher crosslink densities than those containing A380. ILs significantly shortened (by approximately 14–31 min) the t_95_ of SBR compounds filled with A380. The most pronounced effect was achieved for BmiBr, which reduced the t_95_ from 42 min to 11 min. The same IL decreased the t_95_ of the VN3-filled SBR compound, whereas BmpyrBr and BmpipBr did not shorten the t_95_ of SBR containing VN3. Regardless of the silica used, ILs had a beneficial influence on the vulcanization efficiency, significantly increasing the crosslink density of the vulcanizates. The highest crosslink density was demonstrated by the vulcanizates with BmiBr. The beneficial influence of ILs on the t_95_ and crosslink density of the silica-filled vulcanizates was probably due to ILs’ adsorption on the silica surface, which reduced its ability to adsorb curatives. ILs such as silane coupling agents were reported to adsorb onto the silica surface, decreasing its ability to adsorb the curing system and thus enhancing the crosslink density of vulcanizates [24]. Moreover, ILs, due to their catalytic activity in interfacial reactions, such as crosslinking reactions, may accelerate vulcanization and increase its efficiency [50,51]. The beneficial effect of ILs on the crosslink density of vulcanizates was also confirmed by other researchers [52,53,54].

**Figure 5:**
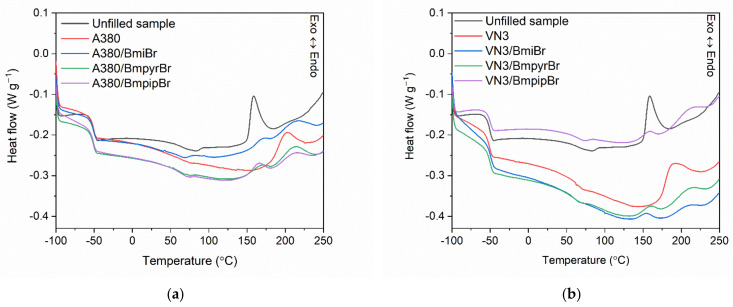
Differential scanning calorimetry (DSC) curves of SBR compounds filled with: (**a**) A380; (**b**) VN3.

Having established the influence of the specific surface area of silica and the addition of ILs on the rheometric properties of SBR compounds and crosslink density of the vulcanizates, then their impact on the enthalpy and temperature of crosslinking reactions was explored by employing differential scanning calorimetry (DSC). The results for SBR compounds are presented in Figure 5 (DSC curves) and summarized in Table 5.

Analyzing the DSC curves of SBR compounds, the transition of the SBR elastomer from the glassy state to the elastic region upon heating is visible as a step produced by the change in the heat capacity (ΔC_p_) of the material. The glass transition temperature (T_g_), which corresponds to the midpoint of the above-mentioned inflection on the DSC curves, is crucial for setting the operating temperature range of rubber products. Hence, the first phase transition visible on the DSC curves was identified as the glass transition of the SBR elastomer. Regardless of the type of silica and the structure of ILs used, SBR exhibited a similar T_g_ in the range of −51.9 °C to −50.3 °C (the differences were in the range of the measurement standard deviation). The type of silica and ILs did not significantly affect the ∆C_p_ during glass transition. 

The crosslinking of the reference unfilled SBR compound proceeded as a one-step exothermic process in the temperature range of 149–188 °C, so much narrower than for rubber compounds containing silica. The enthalpy (∆H) of crosslinking was approximately 9.0 J/g. The type of silica, i.e., the specific surface area, affected the crosslinking temperature and enthalpy. Regarding rubber compounds without ILs, SBR filled with A380 demonstrated a 27 °C higher onset crosslinking temperature compared to the unfilled SBR and an approximately 9 °C higher onset crosslinking temperature than that of VN3-filled rubber. Therefore, the curing system was less effective compared to the unfilled rubber compounds due to the adsorption of the curatives on the silica surface, which was particularly evident for A380 with a remarkably higher specific surface area. Moreover, regardless of the silica used, the crosslinking of filled rubber compounds occurred in a much wider range of temperatures compared to unfilled SBR. The endset crosslinking temperature was 188 °C and approximately 240 °C for the unfilled SBR compound and those containing silica, respectively. This may result from the fact that the filler network hinders the diffusion of heat and components of the crosslinking system through the elastomer matrix during vulcanization. It is worth noting that ILs, regardless of their structure, caused a significant decrease in the onset crosslinking temperature of all silica-filled rubber compounds to a value similar to that of the unfilled SBR. Consequently, DSC results confirmed the beneficial influence of ILs on the vulcanization of SBR compounds filled with A380 or VN3 silica. Therefore, the application of these ILs may allow for crosslinking SBR at lower temperatures than the commonly used 160 °C. Contrary to rubber compounds without ILs, the crosslinking of SBR containing these additives occurred as a two-step process with less heat release. Thus, some post-curing reactions were expected at the second step, e.g., the cross-polymerization of butadiene segments in the SBR macromolecules, the fragmentation of elastomer chains, and the creation of volatile products of their thermal degradation [55]. A similar influence of ILs on crosslinking reactions was noted for ethylene–propylene–diene elastomer composites [56,57].

### 3.4. Effect of Silica and Ionic Liquids on Tensile Properties and Hardness of SBR Composites 

**Figure 6:**
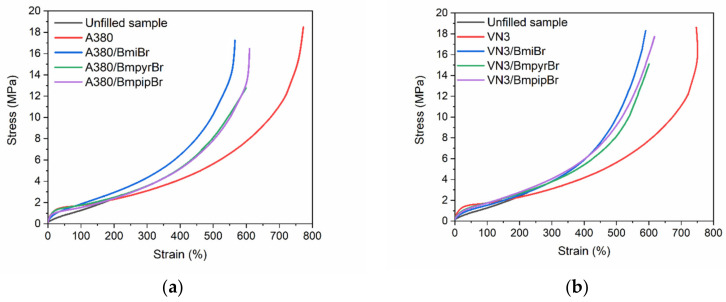
Stress–strain curves of SBR vulcanizates filled with: (**a**) A380; (**b**) VN3. Analyzing the data collected in Table 6, no significant influence of the specific surface area of silica on the stress at a relative elongation of 100% and 300% (Se_100_, Se_300_, respectively) was observed. However, due to the incorporation of the filler, silica-filled SBR vulcanizates exhibited slightly higher Se_100_ compared to the unfilled sample. Addition of ILs, especially BmiBr, increased the Se_300_ by approximately 1 MPa as compared to the vulcanizates without ILs. This resulted from the higher crosslink density of the vulcanizates containing ILs.

As was already mentioned, specific surface area is one of the main parameters affecting the activity and the reinforcing effect of filler. Thus, we studied the influence of silica’s specific surface area on the mechanical properties and hardness of the SBR vulcanizates. Moreover, as confirmed earlier, the type of silica and ILs altered the crosslink density of the vulcanizates. Since the crosslink density significantly affects the mechanical properties and hardness of vulcanized rubber [58], the influence of the silica and ILs used on the performance of SBR is expected. The results are given in Table 6, whereas the stress–strain curves are presented in Figure 6. 

As expected, the specific surface area of silica and the addition of ILs strongly affected the tensile strength (TS) and elongation at break (E_b_) of SBR vulcanizates (Table 6, Figure 6). The unfilled SBR vulcanizate reached a TS of 2.4 MPa and an E_b_ of approximately 222%, so it was characterized by poor mechanical strength. Both silicas showed the reinforcing effect towards the SBR elastomer and allowed us to achieve vulcanizates with a TS of approximately 19 MPa. Thus, no significant influence of the silica’s specific surface area on the TS was observed, but it should be taken into account that the VN3-filled vulcanizate exhibited a remarkably higher crosslink density compared to the A380-containing elastomer, which facilitated the TS as well. ILs, especially BmpyrBr, deteriorated the TS of SBR vulcanizates, especially those filled with A380. Among the IL-containing elastomers, the highest TS was shown by the vulcanizates with BmiBr (18.7 MPa for VN3 and 18.2 MPa for A380, respectively), so those with the highest crosslink density. On the other hand, due to the lowest crosslink density, vulcanizates with BmpyrBr exhibited a TS that was approximately 4 MPa (VN3) and 6 MPa (A380) lower compared to the vulcanizates without ILs.

Regardless of the silica used, the E_b_ of the vulcanizates was significantly higher (749% for A380 and 690% for VN3, respectively) compared to the unfilled elastomer (E_b_ of 222%). The addition of ILs reduced the E_b_ of SBR vulcanizates by approximately 150%, thus decreasing the elastomer flexibility. This was also due to the increased crosslink density of the vulcanizates with ILs compared to those containing only a filler. Regarding A380 silica, the E_b_ of the IL-containing vulcanizates was in the range of 560–600%, whereas the E_b_ of VN3-filled SBR ranged from 587% to 600%.

The addition of silica significantly altered the hardness of the vulcanizates. The influence of the silica specific surface area was also noticed. The unfilled SBR vulcanizate exhibited a hardness of 43 Shore A. Both silicas enhanced the hardness of SBR vulcanizates by approximately 20 Shore A compared to the unfilled sample. This was due to the reinforcement effect of the filler and significantly reduced mobility of elastomer chains by the filler network created inside the elastomer matrix. As expected, A380 silica had the most pronounced effect, increasing the hardness to 63 Shore A. The VN3-filled vulcanizate showed a hardness of 58 Shore A, but it should be mentioned that this vulcanizate exhibited a significantly higher crosslink density compared to the A380-filled elastomer, which contributed to the hardness as well. ILs caused a further increase in the hardness of the vulcanizates (by 4–7 Shore A for A380 and by 3–5 Shore A for VN3, respectively) due to the significant increase in their crosslink density. As expected, the highest hardness was determined for the vulcanizates with BmiBr, which exhibited the highest crosslink density.

### 3.5. Effect of Silica and Ionic Liquids on Thermo-Oxidative Aging of SBR Composites

**Figure 7:**
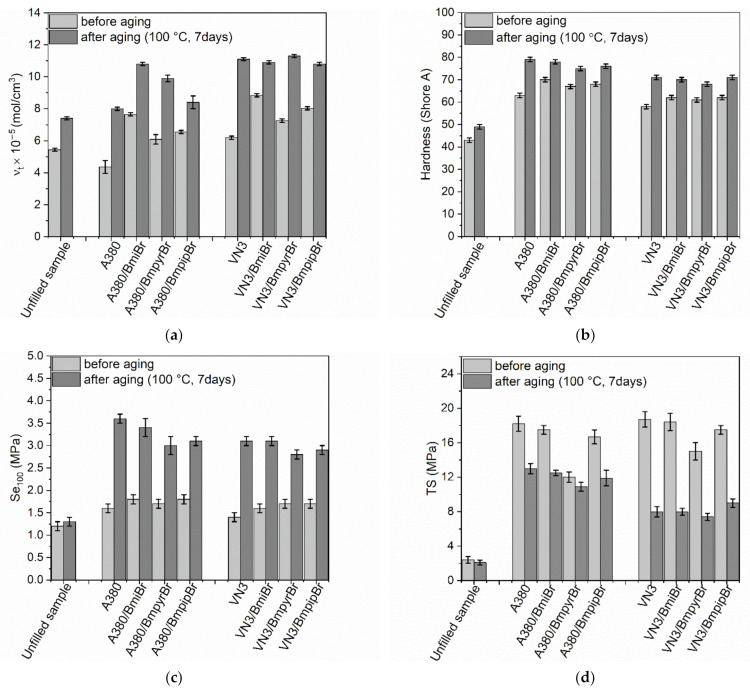

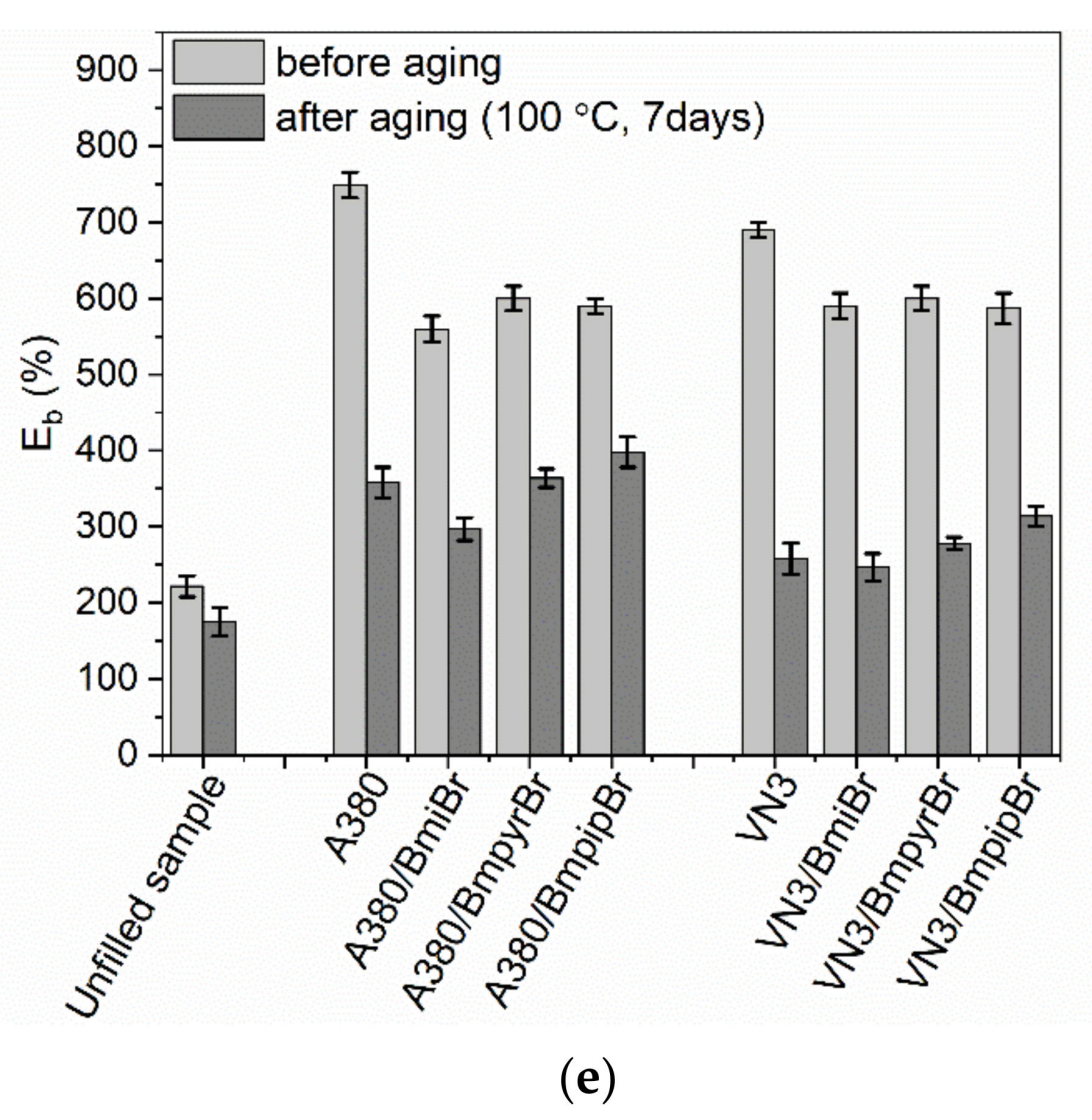
Effect of prolonged thermo-oxidation on the performance and crosslink density of SBR composites filled with silica A380 and VN3: (**a**) crosslink density; (**b**) hardness; (**c**) stress at 100% relative elongation; (**d**) tensile strength; (**e**) elongation at break.

Aging and degradation processes cause extremely serious problems for rubber materials associated with the deterioration of their functional properties as a function of time. There are various methods to improve the resistance of rubber products to thermo-oxidative aging, but the addition of inorganic fillers and antioxidants is the most commonly used method [59]. Therefore, in this work, the influence of the type of silica and ILs on the resistance of SBR composites to thermo-oxidative aging was investigated. The influence of thermo-oxidative aging on the tensile properties, hardness, and crosslink density of the SBR vulcanizates is presented in Figure 7. 

Regardless of the silica type and application of ILs, SBR vulcanizates exhibited higher crosslink densities after prolonged exposure to 100 °C (Figure 7a). A similar influence of thermo-oxidation was achieved for the unfilled sample. According to the literature, it is commonly reported that prolonged exposure to thermo-oxidation causes an increase in the crosslink density of elastomers [41,60,61,62]. The highest increase in the crosslink density after thermo-oxidation was observed for the vulcanizates without ILs and those containing pyrrolidinium salts, i.e., BmpyrBr, which exhibited the lowest crosslink densities before the aging process. 

Since hardness strongly depends on the crosslink density, after thermo-oxidative aging all vulcanizates were characterized with higher hardness compared to non-aged samples (Figure 7b). The hardness of the unfilled benchmark and SBR vulcanizates filled with silica, which were subjected to thermo-oxidative aging, increased by approximately 7–13 Shore A. However, this effect was more pronounced for silica-containing vulcanizates. The type of silica and addition of ILs had no significant effect on the extent of hardness changes after aging. 

Owing to the higher crosslink density, SBR vulcanizates, regardless of their composition, demonstrated higher Se_100_ after thermo-oxidative aging compared to non-aged samples (Figure 7c). The unfilled sample exhibited the smallest increase in Se_100_, whereas the highest SE_100_ increment was achieved for the silica-filled vulcanizates without ILs due to the highest increase in crosslink density upon the aging process.

Regarding the influence of prolonged thermo-oxidation on the TS of the vulcanizates, thermo-oxidative aging slightly reduced the TS of the unfilled benchmark, whereas for the vulcanizates containing silica A380 and VN3 a significantly higher reduction in TS was achieved, i.e., by approximately 5 MPa and 11 MPa for SBR filled with A380 and VN3 silica, respectively (Figure 7d). Regarding SBR vulcanizates with ILs, the highest decrease in TS was exhibited by the VN3-filled vulcanizates with BmiBr and BmpipBr. A significant reduction in the TS of the vulcanizates resulting from thermo-oxidative aging indicated that further crosslinking caused the studied elastomers to be over-crosslinked and thus more susceptible to mechanical stress. Hence, lower strain was sufficient to rupture the vulcanizates compared to non-aged samples. 

As expected, thermo-oxidative aging significantly affected the flexibility of SBR vulcanizates filled with both silicas, causing their E_b_ to decrease compared to the E_b_ of the non-aged vulcanizates (Figure 7e). The highest reduction in E_b_ was observed for the silica-filled vulcanizates without ILs, by approximately 350% compared to the non-aged vulcanizates. On the other hand, the E_b_ of the unfilled vulcanizate was reduced only by 47%. Regarding the SBR vulcanizates with ILs, a higher reduction in E_b_ was determined for VN3-containing vulcanizates (by approximately 300%) than for A380-filled SBR (E_b_ was reduced by approximately 200% compared to that of non-aged vulcanizates). It should be noticed that the decrease in E_b_ fully correlated with the increase in the crosslink density of the vulcanizates; the higher the increase in the crosslink density, the higher the reduction in E_b_ of the vulcanizates due to thermo-oxidative aging.

To facilitate the evaluation of the resistance of SBR vulcanizates to thermo-oxidative aging, the aging coefficient AF was calculated, which is a quantitative measure of the material’s resistance to thermo-oxidative aging. The AF coefficient was determined based on the changes in the tensile properties, i.e., TS and E_b_, of the vulcanizates due to the aging process. The results are listed in Table 7.

Thermo-oxidative aging did not significantly affect the tensile properties of the unfilled SBR vulcanizate. Therefore, it was characterized by an AF of approximately 0.7, so it demonstrated quite good resistance to thermo-oxidative aging. Regarding the vulcanizates without ILs, the type of silica had no significant effect on the resistance of SBR to prolonged thermo-oxidation. On the other hand, regardless of the silica used, filled SBR vulcanizates showed a significantly lower AF compared to the unfilled sample. Thus, they exhibited lower resistance to thermo-oxidative aging. Considering the vulcanizates with ILs, the vulcanizates filled with silica A380 exhibited a higher AF and thus a higher resistance to thermo-oxidation. This was due to the slightly smaller changes in the crosslink density of the A380-containing vulcanizates, and consequently their mechanical properties compared to those of non-aged samples.

### 3.6. Effect of Silica and Ionic Liquids on Thermal Stability of SBR Cmposites

**Figure 8:**
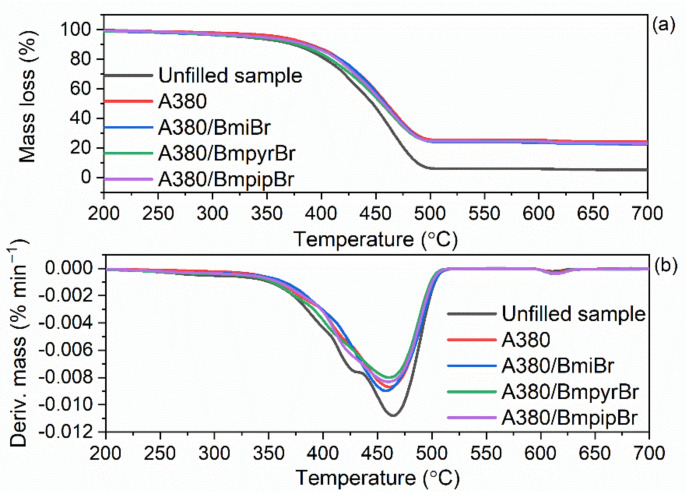
Thermogravimetric (TG) and derivative thermogravimetric (DTG) curves of SBR composites filled with silica A380: (**a**) TG curves; (**b**) DTG curves.

**Figure 9:**
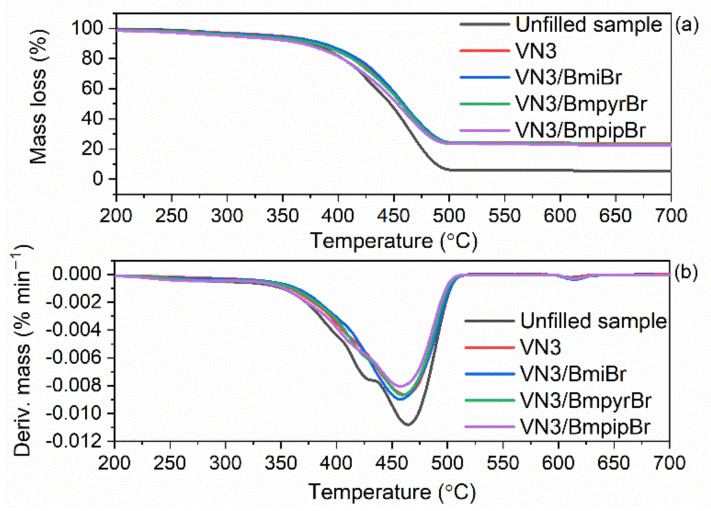
TG and DTG curves of SBR composites filled with silica VN3: (**a**) TG curves; (**b**) DTG curves.

As was discussed earlier, the ILs and silicas applied in this study exhibited different thermal stabilities, which may affect the thermal stability of the SBR vulcanizates. The thermal performance of elastomer composites is known to depend on the thermal stability of both the components of rubber compounds and the rubber matrix. Therefore, the impact of ILs and silica type on the thermal stability of SBR vulcanizates was examined by applying thermogravimetry (TG), and the results are presented in Table 8 and in Figure 8 and Figure 9.

The thermal decomposition of the unfilled SBR vulcanizate started at a temperature of approximately 331 °C (Table 8). As expected, the type of the silica and the addition of ILs substantially affected the onset decomposition temperature (T_5%_) of SBR vulcanizates, but had no meaningful impact on the T_DTG_, which is the temperature of the maximum mass loss rate and was in the range of 469–481 °C. Regarding the vulcanizates without ILs, the addition of the silica A380 and VN3 improved the thermal stability of SBR composites compared to the unfilled one. The A380-filled vulcanizate exhibited a T_5%_ of 363 °C, while VN3-containing SBR was characterized by a T_5%_ of 347 °C, so approximately 16 °C lower compared to that of the A380-filled elastomer. It should be noted that the T_5%_ of the A380-filled vulcanizate was approximately 32 °C higher as compared to the unfilled sample. The highest thermal stability of the A380-filled vulcanizates was due to the network created by silica A380 nanoparticles dispersed in the elastomer matrix, which impedes the diffusion of volatile products and gases released during thermal decomposition through the material, thus improving the thermal stability [63]. Regardless of the type of silica used, ILs deteriorated the thermal stability of SBR vulcanizates as compared to vulcanizates without ILs. This resulted from the thermal decomposition of ILs, which, as was discussed earlier, started at a temperature of approximately 240 °C. However, the thermal stability of most vulcanizates containing ILs was still higher as compared to the unfilled sample. The least effect on the thermal stability of SBR vulcanizates was exhibited by BmiBr, which reduced T_5%_ by approximately 6–8 °C compared to the vulcanizates without ILs. On the other hand, BmpyrBr reduced T_5%_ by 26–28 °C. The same effect was achieved in our previous work for NR vulcanizates [17], and thus we confirmed that BmiBr-containing elastomers are characterized by a higher thermal stability than those with BmpyrBr, which is due to the higher thermal stability of pure BmiBr as compared with BmpyrBr.

Regarding the unfilled sample, the mass loss in the temperature range of 25–600 °C was approximately 94% and resulted from the pyrolysis of elastomer and organic components of the rubber compounds, i.e., vulcanization accelerators. Thus, for the silica-filled SBR, this mass loss was slightly higher for the vulcanizates containing ILs compared to those without ILs, since ILs also decomposed in this temperature range. Owing to the incorporation of silica into rubber composites, the mass loss in the temperature range of 25–600 °C was approximately 20% lower compared to the unfilled sample. The mass loss in the temperature range of 600–800 °C was determined in an air atmosphere. It corresponds to the combustion of the residue after the pyrolysis of the organic additives and elastomer. Therefore, it was slightly higher for the IL-containing composites compared to the unfilled sample and those without ILs. Regarding the unfilled vulcanizate, the residue at 800 °C of approximately 5.5% resulted from ash and zinc oxide, which was used as a vulcanization activator. In the case of the silica-filled SBR, this residue was in the range of 22.7–24.5% and contained silica in addition to ash and zinc oxide.

### 3.7. Effect of Silica and Ionic Liquids on Dynamic Mechanical Properties of SBR Composites

**Figure 10:**
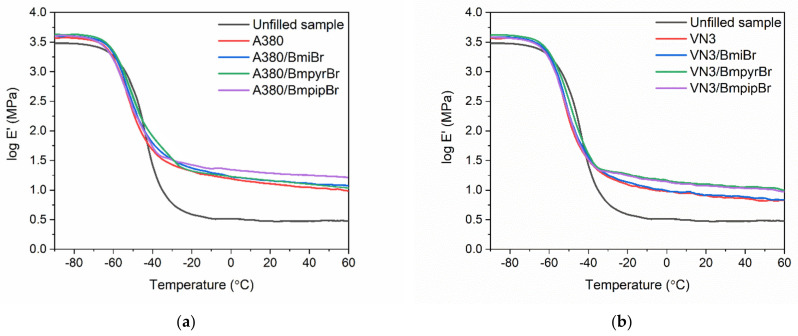
Storage modulus (E’) curves versus temperature of SBR composites filled with silica: (**a**) A380; (**b**) VN3.

**Figure 11:**
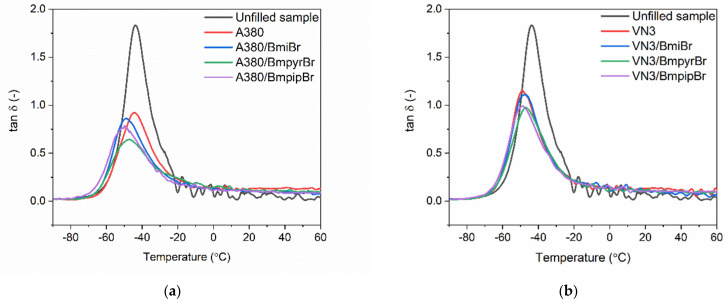
Loss factor (tan δ) curves versus temperature of SBR composites filled with silica: (**a**) A380; (**b**) VN3.

Having explored that the specific surface area of silica and the addition of ILs affected the tensile properties of the vulcanizates measured in static conditions, we then analyzed the influence of the silica type and structure of ILs on the viscoelastic properties of SBR vulcanizates and therefore their ability to dampen vibrations. Dynamic mechanical analysis (DMA) was employed to perform the measurements of viscoelastic properties of SBR vulcanizates as a function of temperature. Hereby, the influence of the applied additives on the glass transition of the SBR elastomer and its viscoelastic properties in the rubbery elastic region was established. The DMA curves of SBR vulcanizates are plotted in Figure 10 and Figure 11, whereas the results are summarized in Table 9.

Applying silica fillers regardless of their specific surface area affected the storage modulus (E’) of SBR measured as a function of temperature. Silica-filled SBR exhibited higher E’ values both in the glassy state and in the rubbery elastic region as compared to the unfilled vulcanizate. This might be attributed to the higher stiffness of the filled composites resulting from the reduced elastomer chain mobility by the filler network in comparison with the unfilled elastomer. A similar influence of silica on E’ values compared to the unfilled vulcanizate was notified by Prasertsri et al. [64] for fumed and precipitated silica applied as a filler of natural rubber composites. The specific surface area of silica did not significantly affect the E’ of SBR composites or their ability to store mechanical energy during the stress period. This was also in line with Prasertsri’s [64] observations.

Analyzing the DMA curves of all vulcanizates plotted in Figure 11, only the glass transition of the SBR elastomer was observed as the maximum of the mechanical loss factor (tan δ) peak in the DMA curve. Thus, the temperature at which the maximum of the tan δ peak occurs in the DMA curve corresponds to the glass transition temperature (T_g_) of SBR. The T_g_ of SBR determined for the unfilled vulcanizate was approximately −46.6 °C. Regarding the effect of the silica type and IL structure on the T_g_ of the SBR elastomer, no remarkable influence was observed considering the measurement error. Thus, the results of DMA analysis correlate well with the data obtained by DSC (Table 5). The highest value of tan δ at T_g_ (tan δ_Tg_ in Table 9) was exhibited by the unfilled reference vulcanizate. It was of approximately 1.8 and resulted from the highest flexibility of the unfilled vulcanizate as compared with SBR filled with silica. The addition of both silicas, especially A380, significantly reduced the height of the tan δ peak owing to the decreased elastomer chain mobility by the filler network in comparison with the unfilled elastomer [65]. The addition of fillers, which created their own network inside the crosslinked elastomer matrix, increased the stiffness and therefore reduced the flexibility of the SBR vulcanizates, and hence the values of tan δ at T_g_. The same influence of silica on the damping properties and consequently tan δ of vulcanizates compared to the unfilled rubber was reported by Yoon et al. for the composites of SBR with polybutadiene rubber [66]. Considering the tan δ at 25 °C and 50 °C, i.e., in the rubbery elastic region, the specific surface area of silica and the structure of ILs did not significantly affect the damping properties of the SBR elastomer. However, introducing a filler to the elastomer matrix caused a significant increase in tan δ at 25 °C and 50 °C as compared to the reference unfilled sample, which was due to the much higher stiffness of the vulcanizates containing silica. The analysis of tan δ curves allowed us to confirm that the damping properties of the studied composites are stable in the rubbery elastic region since tan δ values did not significantly change in the temperature range of 25–60 °C.

## 4. Conclusions

Introducing a silica filler to SBR compounds prolonged the optimal vulcanization time and reduced the crosslink density compared to the unfilled benchmark, which was due to the adsorption of curatives onto the filler surface, especially in the case of A380 silica with a significantly higher specific surface area compared to VN3. Thus, vulcanizates filled with A380 exhibited a lower crosslink density than that of the VN3-filled SBR. ILs, especially BmiBr, beneficially affected the optimal vulcanization time and crosslink density of SBR composites, which was the most important for the A380-filled SBR. The advantageous effect of ILs on the cure characteristics of SBR compounds probably resulted from the adsorption of ILs on the silica surface, which limited the adsorption of the curatives. 

The specific surface area of silica and the structure of ILs have an impact on the tensile strength and elongation at break of SBR vulcanizates. Both tested silicas showed a reinforcing effect towards SBR. The VN3-filled vulcanizates exhibited slightly better mechanical performance due to their considerably higher crosslink density compared with A380-filled SBR. Regardless of the silica used, ILs slightly deteriorated the tensile strength of SBR vulcanizates, especially those filled with silica A380. This resulted from the increase in the crosslink density of SBR vulcanizates under the influence of ILs. The type of silica and ILs have a significant effect on the hardness of SBR vulcanizates, causing it to increase compared to unfilled SBR. Applying silica, especially VN3, deteriorated the resistance of SBR vulcanizates to thermo-oxidative aging compared to the unfilled composite, whereas ILs improved the aging resistance of the A380-filled SBR but had no considerable influence on the aging properties of the VN3-containing composites. SBR vulcanizates filled with A380 and VN3 silica and containing ILs exhibited acceptable damping properties. Additionally, an improved thermal stability was achieved due to the filler’s network created in the crosslinked elastomer matrix.

The performed studies proved that ILs, especially BmiBr, showed a beneficial influence on the vulcanization parameters of SBR compounds, as well as on the crosslink density and performance of the vulcanizates; thus, they can be successfully used to enhance the properties of elastomer compounds containing silica with various specific surface areas.

## Figures and Tables

**Table 1:** Ionic liquids (ILs) with bromide anion used in the styrene–butadiene rubber (SBR) compounds.

Name	Abbreviation	CAS Number	Purity (%)	Water Content (wt.%)
1-butyl-3-methylimidazolium bromide	BmiBr	85100-77-2	≥99.0%	<0.5%
1-butyl-1-methylpyrrolidinium bromide	BmpyrBr	93457-69-3	≥99.0%	≤0.5%
1-butyl-1-methylpiperidinium bromide	BmpipBr	94280-72-5	≥99.0%	0.5%

**Table 2:** General recipes of styrene–butadiene rubber (SBR) reference compounds, parts per hundred of rubber (phr); (MBT, 2-mercaptobenzothiazole; CBS, N-cyclo-hexyl-2-benzothiazolesulfenamide).

Ingredient, phr	Unfilled Sample (R0)	SBR/A380 (R1)	SBR/VN3 (R2)
SBR	100	100	100
Sulfur	2	2	2
ZnO	5	5	5
MBT	1	1	1
CBS	1	1	1
Aerosil 380	₋	30	₋
Ultrasil VN3	₋	₋	30

**Table 3:** General recipes of SBR compounds containing ionic liquids (phr); (BmiBr, 1-butyl-3-methylimidazolium bromide; BmpyrBr, 1-butyl-1-methylpyrrolidinium bromide; BmpipBr, 1-butyl-1-methylpiperidinium bromide).

Ingredient, phr	SBR/BmiBr (IL1–IL2)	SBR/BmpyrBr (IL3–IL4)	SBR/BmpipBr (IL5–IL6)
SBR	100	100	100
Sulfur	2	2	2
ZnO	5	5	5
MBT	1	1	1
CBS	1	1	1
Silica *	30	30	30
BmiBr	3	₋	₋
BmpyrBr	₋	3	₋
BmpipBr	₋	₋	3

* Silica, Aerosil 380 or Ultrasil VN3, alternatively: IL1, IL3, IL5—rubber compounds filled with Aerosil 380; IL2, IL4, IL6—rubber compounds filled with Ultrasil VN3.

**Table 4:** Cure characteristics at 160 °C of SBR compounds filled with A380 and VN3 silica and crosslink density of vulcanizates (*S_min_*, minimum torque; *S_max_*, maximum torque; ∆*S*, torque increase; t_05_, scorch time; t_95_, optimal vulcanization time; standard deviations: *S_min_* ± 0.3 dNm, *S_max_* ± 1.3 dNm, Δ*S* ± 1.5 dNm, t_05_ ± 0.1 min, t_95_ ± 0.4 min.; ν_t_ ± 0.3 × 10^−5^ mol/cm^3^).

Compounds	*S_min_* (dNm)	*S_max_* (dNm)	∆*S* (dNm)	t_05_ (min)	t_95_ (min)	$\nu_{t}\times 10{-}^{5}$ (mol/cm^3^)
Unfilled sample	0.7	8.1	7.4	1	3	5.44
Aerosil A380						
A380	4.5	24.5	20.0	2	42	4.36
A380/BmiBr	4.1	24.3	19.2	1	11	7.65
A380/BmpyrBr	4.3	22.0	17.8	1	28	6.09
A380/BmpipBr	3.7	22.2	18.5	1	21	6.55
Ultrasil VN3						
VN3	2.3	18.0	15.7	2	10	6.19
VN3/BmiBr	1.7	17.0	15.5	1	3	8.84
VN3/BmpyrBr	1.9	16.5	14.6	1	17	7.26
VN3/BmpipBr	2.3	17.2	14.5	1	15	8.03

**Table 5:** Differential scanning calorimetry (DSC) of SBR compounds filled with A380 and VN3 silica (T_g_, glass transition temperature; ∆C_p_, heat capacity; T_onset_, the onset temperature of crosslinking; T_endset_, the endset temperature of crosslinking; ∆H, enthalpy of crosslinking).

Compounds	T_g_ (°C)	∆C_p_ (J/g × K)	T_onset_ (°C)	T_endset_ (°C)	∆H (J/g)
Unfilled sample	−51.1 ± 0.9	0.40 ± 0.10	149 ± 2	188 ± 2	9.0 ± 1.5
Aerosil 380					
A380	−51.5 ± 1.0	0.33 ± 0.09	176 ± 3	235 ± 3	8.6 ± 1.4
A380/BmiBr	−51.2 ± 0.9	0.33 ± 0.08	145 ± 1	243 ± 1	7.1 ± 1.3
A380/BmpyrBr	−51.9 ± 1.1	0.35 ± 0.09	148 ± 2	241 ± 2	7.3 ± 1.4
A380/BmpipBr	−51.5 ± 1.0	0.33 ± 0.08	146 ± 3	239 ± 3	5.9 ± 1.5
Ultrasil VN3					
VN3	−50.9 ± 0.9	0.34 ± 0.09	167 ± 1	235 ± 1	12.7 ± 1.5
VN3/BmiBr	−50.4 ± 0.8	0.36 ± 0.07	137 ± 1	230 ± 1	3.0 ± 1.5
VN3/BmpyrBr	−50.3 ± 0.9	0.34 ± 0.09	140 ± 2	235 ± 2	4.8 ± 1.4
VN3/BmpipBr	−50.6 ± 0.9	0.32 ± 0.10	140 ± 2	235 ± 2	4.7 ± 1.3

**Table 6:** Mechanical properties and hardness of SBR vulcanizates filled with A380 and VN3 silica (Se_100_, stress at 100% relative elongation; Se_300_, stress at 300% relative elongation; TS, tensile strength; E_b_, elongation at break; H, hardness).

SBR Vulcanizates	Se_100_ (MPa)	Se_300_ (MPa)	TS (MPa)	E_b_ (%)	H (Shore A)
Unfilled sample	1.2 ± 0.1	-	2.4 ± 0.4	222 ± 14	43 ± 1
Aerosil 380					
A380	1.6 ± 0.1	2.5 ± 0.2	18.2 ± 0.9	749 ± 17	63 ± 1
A380/BmiBr	1.8 ± 0.1	4.0 ± 0.2	17.5 ± 0.5	560 ± 17	70 ± 1
A380/BmpyrBr	1.7 ± 0.1	3.5 ± 0.1	12.0 ± 0.6	600 ± 16	67 ± 1
A380/BmpipBr	1.8 ± 0.1	3.7 ± 0.1	16.7 ± 0.8	590 ± 10	68 ± 1
Ultrasil VN3					
VN3	1.4 ± 0.1	2.8 ± 0.1	18.7 ± 0.9	690 ± 10	58 ± 1
VN3/BmiBr	1.6 ± 0.1	3.9 ± 0.1	18.4 ± 1.0	590 ± 17	63 ± 1
VN3/BmpyrBr	1.7 ± 0.1	3.7 ± 0.3	15.0 ± 1.0	600 ± 16	61 ± 1
VN3/BmpipBr	1.7 ± 0.1	3.8 ± 0.3	17.5 ± 0.5	587 ± 20	62 ± 1

**Table 7:** Thermo-oxidative aging coefficient (AF) of SBR vulcanizates filled with silica A380 and VN3 (SD: AF ± 0.1).

SBR Vulcanizates	AF (-)
Unfilled sample	0.7
Aerosil 380	
A380	0.3
A380/BmiBr	0.4
A380/BmpyrBr	0.6
A380/BmpipBr	0.5
Ultrasil VN3	
VN3	0.2
VN3/BmiBr	0.2
VN3/BmpyrBr	0.2
VN3/BmpipBr	0.3

**Table 8:** Onset temperature of thermal decomposition (T_5%_), DTG peak temperature (T_DTG_), and total mass loss (∆m) during decomposition of SBR composites filled with silica A380 and VN3.

SBR Vulcanizates	T_5%_ (°C)	T_DTG_ (°C)	∆m_25–600 °C_ (%)	∆m_600–800 °C_ (%)	Residue at 800 °C (%)
Unfilled sample	331 ± 1	481 ± 1	94.1 ± 1.2	0.4 ± 1.2	5.5 ± 1.0
Aerosil 380					
A380	363 ± 1	478 ± 1	74.8 ± 1.1	0.7 ± 1.1	24.5 ± 0.8
A380/BmiBr	357 ± 1	469 ± 1	74.9 ± 1.0	1.4 ± 1.0	23.7 ± 0.7
A380/BmpyrBr	337 ± 2	477 ± 2	75.3 ± 1.3	1.2 ± 1.3	23.5 ± 0.9
A380/BmpipBr	347 ± 1	477 ± 1	75.4 ± 1.3	1.2 ± 1.3	23.4 ± 0.9
Ultrasil VN3					
VN3	347 ± 1	479 ± 1	75.7 ± 1.0	0.8 ± 1.0	23.5 ± 0.8
VN3/BmiBr	339 ± 1	474 ± 1	76.1 ± 0.9	1.2 ± 0.9	22.7 ± 0.8
VN3/BmpyrBr	319 ± 2	477 ± 2	75.9 ± 1.3	1.1 ± 1.3	23.0 ± 0.9
VN3/BmpipBr	301 ± 1	475 ± 1	76.3 ± 1.2	1.0 ± 1.2	22.7 ± 0.9

**Table 9:** Glass transition temperature (T_g_) determined by dynamic mechanical analysis (DMA) and mechanical loss factor (tan δ) of SBR composites filled with silica A380 and VN3.

SBR Vulcanizates	T_g_ (°C)	Tan δ_Tg_ (-)	Tan δ_25 °C_ (-)	Tan δ_50 °C_ (-)
Unfilled sample	−46.6 ± 1.1	1.8 ± 0.2	0.04 ± 0.02	0.03 ± 0.02
Aerosil A380				
A380	−49.4 ± 1.2	0.9 ± 0.1	0.12 ± 0.01	0.13 ± 0.01
A380/BmiBr	−49.1 ± 1.0	0.9 ± 0.1	0.10 ± 0.01	0.08 ± 0.01
A380/BmpyrBr	−47.4 ± 1.2	0.6 ± 0.2	0.11 ± 0.02	0.10 ± 0.02
A380/BmpipBr	−50.0 ± 1.2	0.8 ± 0.2	0.10 ± 0.01	0.09 ± 0.01
Ultrasil VN3				
VN3	−48.9 ± 1.0	1.2 ± 0.1	0.14 ± 0.02	0.10 ± 0.02
VN3/BmiBr	−49.2 ± 1.0	1.1 ± 0.1	0.10 ± 0.01	0.10 ± 0.01
VN3/BmpyrBr	−47.2 ± 1.1	1.0 ± 0.2	0.11 ± 0.01	0.10 ± 0.01
VN3/BmpipBr	−48.8 ± 1.1	1.0 ± 0.1	0.10 ± 0.02	0.10 ± 0.02

## Data Availability

The data presented in this study are available on request from the corresponding author.
